# A fast, sensitive and fluorescent LPMO activity assay

**DOI:** 10.3389/fmicb.2023.1128470

**Published:** 2023-03-14

**Authors:** Johan Ø. Ipsen, Katja S. Johansen, Søren Brander

**Affiliations:** Department of Geosciences and Natural Resource Management, University of Copenhagen, Copenhagen, Denmark

**Keywords:** fluorescin, redox activity, copper enzyme, peroxide, LPMO, enzyme assay

## Abstract

Lytic polysaccharide monooxygenases (LPMOs) are industrially relevant enzymes that utilize a copper co-factor and an oxygen species to break down recalcitrant polysaccharides. These enzymes are secreted by microorganisms and are used in lignocellulosic refineries. As such, they are interesting from both the ecological/biological and industrial perspectives. Here we describe the development of a new fluorescence-based kinetic LPMO activity assay. The assay is based on the enzymatic production of fluorescein from its reduced counterpart. The assay can detect as little as 1 nM LPMO with optimized assay conditions. Furthermore, the reduced fluorescein substrate can also be used to identify peroxidase activity as seen by the formation of fluorescein by horseradish peroxidase. The assay was shown to work well at relatively low H_2_O_2_ and dehydroascorbate concentrations. The applicability of the assay was demonstrated.

## 1. Introduction

Lytic polysaccharide monooxygenases (LPMOs) are important industrial enzymes for the production of lignocellulosic ethanol (Johansen, 2016). LPMOs act in synergy with glycoside hydrolases to degrade recalcitrant polymers such as cellulose and chitin (Ipsen et al., 2021). LPMOs that cleave starch, hemicellulose, and pectin have also been described (Frommhagen et al., 2015; Lo Leggio et al., 2015; Sabbadin et al., 2021). Apart from their industrial application, LPMOs are of great interest because they are required in biological processes such as allorecognition in filamentous fungi (Detomasi et al., 2022) and the pathogenicity of some bacteria and fungi that cause human disease (Askarian et al., 2021; Probst et al., 2022). They are mononuclear copper enzymes that require a reductant and an oxygen-containing co-substrate for the cleavage of glucosidic bonds (Frommhagen et al., 2015). LPMO activity can be determined using lengthy and laborious HPLC methods coupled to either amperometric detection or mass spectrometers (Quinlan et al., 2011; Westereng et al., 2013). Furthermore, LPMO action is often probed by their synergy with glycoside hydrolases (Scott et al., 2016), followed by determination of free sugar concentration by a reducing sugars colorimetric assay (Tokin et al., 2020). In the absence of polysaccharide substrate, LPMOs may catalyze a futile reduction of oxygen with concurrent consumption of reductant, i.e., ascorbate, which produces H_2_O_2_. This futile cycle has been used as a proxy for LPMO activity using ascorbate or Amplex Red as the probe (Isaksen et al., 2014; Brander et al., 2020). While LPMOs can produce H_2_O_2_, they can also consume it (Bissaro et al., 2017), and this has been used to develop an assay based on H_2_O_2_-dependent dimerization of 2,6-dimethoxyphenol (DMP) into the chromophore coerulignone (Breslmayr et al., 2018). Another LPMO assay was recently developed based on dehydroascorbate-dependent oxidation of reduced phenolphthalein (rPh)(Brander et al., 2021). This assay can be viewed as an LPMO-optimized version of the Kastle-Meyer presumptive blood test used in forensic science (Cox, 1991). Brander and co-workers hypothesized that the central carbon in the triphenol methyl structure has a C-H bond that can be broken by LPMOs in a reaction that mimics the initial deprotonation of carbohydrates, leading to cleavage of glycosidic bonds. One shortcoming of the phenolphthalein (Ph)-based assay is that it requires color development in alkaline conditions, and thus the reaction must be stopped by addition of base to assess the enzyme activity. A continuous LPMO assay would clearly be advantageous as it requires less handling and fewer samples. Such a fast and specific assay could simplify screening for new or engineered LPMOs and accelerate biochemical characterization.

**Figure 1:**
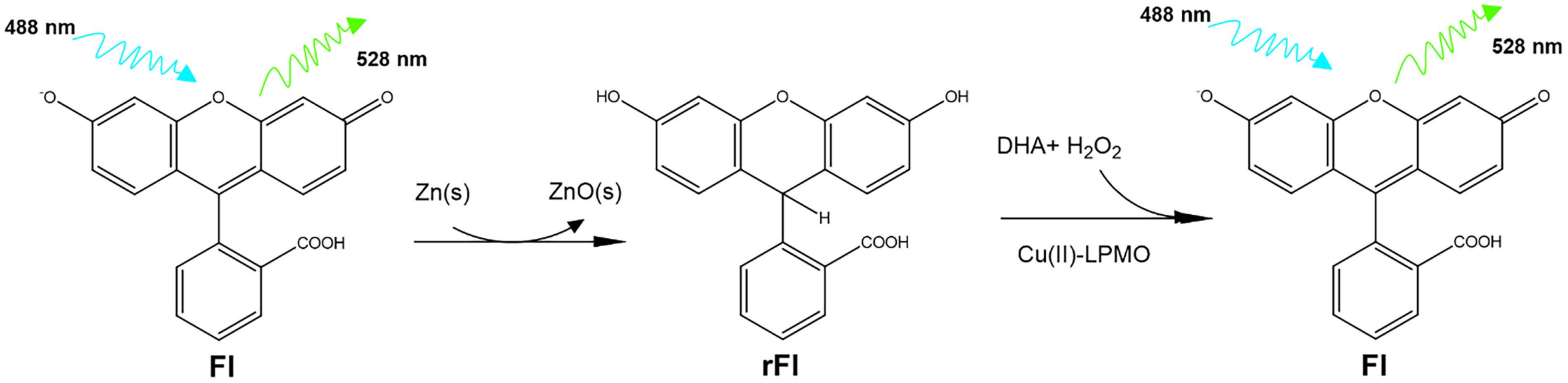
Reaction scheme for the interconversion of Fl and rFl. First Fl is reduced by boiling in NaOH with zinc powder. Residual zinc and zinc oxide is removed by filtration and the resulting rFl can then be re-oxidized by the action of LPMO.

We have investigated fluorescein (Fl) as an alternative to Ph as an LPMO activity probe. Fl shares the xanthene structure with Amplex Red and the triphenol methane structure with Ph. Due to these similarities, we hypothesized that reduced fluorescein (rFl) could be used as an LPMO substrate. Fl is one of the original synthetic dyes, first synthesized by Baeyer in 1871 and is one of the most fluorescent organic molecules (Baeyer, 1871). To test the usefulness of rFl as an LPMO substrate, we have screened several LPMOs but focus was on optimizing the assay with the well-described AA9 LPMO from *Thermoascus aurantiacus* (*Ta*AA9A) (Quinlan et al., 2011). This is an LPMO which, in combination with glycoside hydrolases, is efficient in the deconstruction of crystalline cellulose fibers, and we have previously used this enzyme as a benchmark to test and develop new assays (Brander et al., 2020, 2021). Figure 1 shows the reaction scheme for the interconversion of Fl and rFl. To the best of our knowledge, this is the first example of a fluorescence-based direct LPMO assay.

## 2. Materials and methods

All materials and chemicals were of the highest purity grade available and were purchased from Merck (Kenilworth, New Jersey, United States) unless otherwise stated. *Thermoascus aurantiacus* AA9 (*Ta*AA9A) UniProtKB: G3XAP7, *Thermothielavioides terrestris* AA9 (*Tt*AA9E) UniProtKB: D0VWZ9, *Neurospora crassa* AA9 (*Nc*AA9E) UniProtKB: Q7RWN7, and *Neurospora crassa* AA9 (*Nc*AA9A) NCBI: XP_959499 were kindly donated by Novozymes A/S. *Lentinus similis* AA9 (*Ls*AA9A) UniProtKB: A0A0S2GKZ1, *Pseudomonas fluorescens* CopC UniProtKB: A0A0D0TME7, *Lactobacillus plantarum* AA10 (*Lp*AA10A) UniProtKB: A0A165NIT9, *Streptomyces coelicolor* AA10 (*Sc*10AA10B) UniProtKB: A3KIM2, *Thermobifida fusca* AA10 (*Tf*AA10A) UniProtKB: Q47QG3, *Bacillus subtilis* laccase (CotA) UniProtKB: H8WGE7 were expressed as described previously (Brander et al., 2014; Brander et al., 2020; Hernández-Rollán et al., 2021; Ipsen et al., 2021). *Synechocystis* flavodoxin (IsiB) UniProtKB: P27319 and *Arabidopsis thaliana* cytochrome P450 reductase (ATR) UniProtKB: F4JQY4 were kindly provided by Dr. Silas Busck Mellor (University of Copenhagen). All enzymes were purified to homogeneity before use. Alcohol dehydrogenase, ascorbate oxidase, superoxide dismutase, galactose isomerase, galactose oxidase, laccase from *Trametes versicolor* and horseradish peroxidase (HRP) were purchased from Merck (Kenilworth, New Jersey, United States). CBHII and β-glucosidase were purchased from Megazyme (Bray, Co. Wicklow, Ireland). The protein concentration of LPMOs were determined by acid hydrolysis followed by separation and quantification of individual amino acids with ion-exchange-HPLC (Barkholt and Jensen, 1989). Flavodoxins were quantified using the absorption of flavin mononucleotide cofactor (445 nm) and laccase using the absorption of proteinaceous Cu(II) (600 nm). Commercial enzymes were used as is.

### 2.1. Preparation of rFl

A stock solution of rFl was prepared by reducing fluorescein with zinc powder. For this, 1 g fluorescein, 6 g zinc powder and 2.1 g NaOH were suspended in 500 ml water and boiled for 12 h as follows. The ingredients were mixed in a glass bottle with a magnetic stir bar with the lid off. A small glass funnel was placed on top of the bottle, and the normal lid was loosely placed over it. The bottle was then placed on a stir plate, the heating plate was set to 120°C, and the mixture was left to slow-boil overnight. Glacial acetic acid (3.6 mL) was then added, and the solution boiled for a further 2 h. The rFl solution was allowed to cool to room temperature, and the zinc oxide was removed by decanting the rFl solution into a new flask. Some fresh zinc powder was added, and the solution was stored at 4°C for over a month without loss of activity. The rFl stock concentration was determined to be 5 mM by first oxidizing a 0.125% solution to completion with HRP and comparing the emission of fluorescence at 528 nm to a standard curve for Fl (Supplementary Figure S1A).

### 2.2. Assay of rFl oxidation

The enzymatic production of fluorescein was determined by fluorescence reading using a Biotek Synergy H1 plate reader (Santa Clara, California, Unites States) with excitation at 488 nm and emission at 528 nm. Assay conditions as follows: 0–1 μM Cu(II)-LPMO, 0–250 μM dehydroascorbic acid (DHA), 6.35 μM rFl and 0–1,000 μM H_2_O_2_. In one instance, ascorbate was added instead of DHA. The reactions were prepared in 100 mM citrate–phosphate buffer, pH 7.25, and H_2_O_2_ was always added last. Samples were prepared in triplicates in black 200 μL microtiter plates (Thermo Fisher Scientific, Waltham, Massachusetts, USA). The plate reader reports relative fluorescence units (RFU) and in assay conditions the conversion factor is *ε* = 181,100 RFU/μM. This number together with the enzyme concentration C is used to calculate turn over frequencies of the enzymatic oxidation of rFl: TOF = rate / (ε x C).

### 2.3. Limit of detection and standard protocol for rFl assay

During the course of this study, the assay conditions for rFl oxidation were optimized in a detailed assay protocol to give final concentrations of 10 μM DHA, 10 μM H_2_O_2_, 6.35 μM rFl. These are the conditions used to determine the assay limit of detection and the protocol goes as follows (96 samples). Step1: Prepare a black microtiter plate with 50 μL protein containing samples. Step 2: Prepare an assay mix of 18 mL 100 mM citrate–phosphate buffer, pH 7.25, 4.8 μL 50 mM DHA, 30 μL 5 mM rFl and 2.4 μL 0.1 M H_2_O_2_. Step 3: Add 150 μL of the assay mix to each protein sample. Step 4: Place the microtiter plate in a spectrophotometer and read the fluorescence intensity (excitation: 488 nm, emission: 528 nm) for 30 min. Prior to each reading, shake the plate using a double-orbital movement for 5 s.

## 3. Results

**Figure 2:**
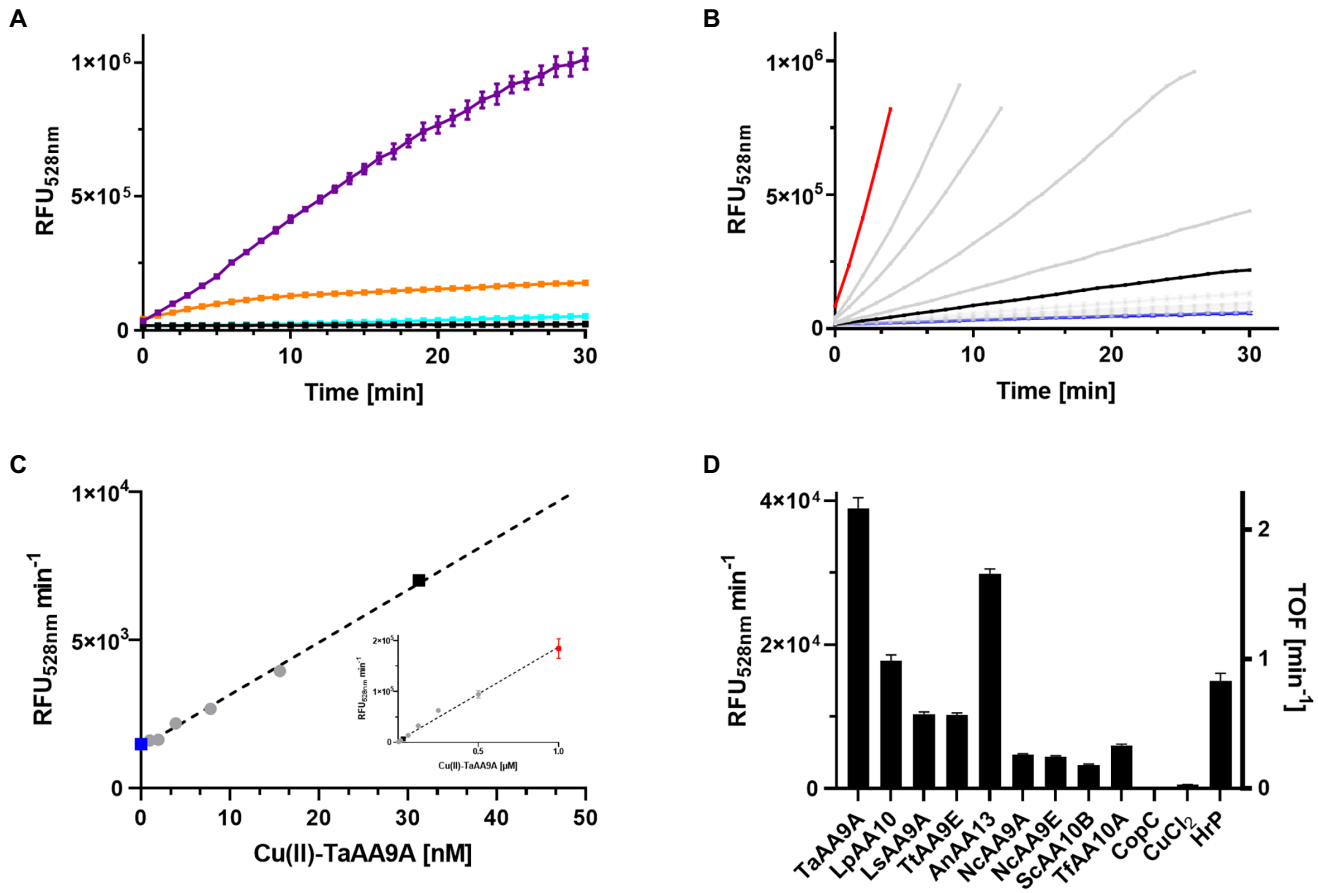
Oxidation of rFl by LPMOs. **(A)** Fluorescence progress curves from incubating samples of 100 nM Cu-*Ta*AA9A (purple), Cu(II)-*Ls*AA9A (orange), CuCl_2_ (cyan), or water (black) with 6.35 μM rFl, 100 μM DHA, and 100 μM H_2_O_2_ in citrate–phosphate buffer at pH 7.25. **(B)** Fluorescence progress curves of similar reactions as in **(A)**, but varying the concentration of Cu(II)-*Ta*AA9A (0–1 μM). The highlighted data are 1 μM (red), 30 nM (black) and 0 (blue) Cu(II)-*Ta*AA9A. **(C)** The initial slopes of the curves in **(B)** are plotted against their enzyme concentration over the range (0–50 nM). The insert shows the complete titration curve from 0–1 μM Cu-TaAA9A. **(D)** A series of LPMOs were tested in the same assay conditions as in **(A)** together with 100 nM CuCl_2_ and 100nM HrP. The initial rates based on these conditions are shown on the left y-axis and the turnover frequency numbers on the right y-axis.

We wanted to test if rFl was an LPMO substrate and used the conditions of the Ph assay as a starting point. However, the rFl and LPMO concentrations should, in theory, be considerably lower due to the sensitivity of fluorometric assays. Furthermore, H_2_O_2_ at an equimolar concentration with DHA was included due to the finding that the *Ls*AA9A activity was strictly dependent on H_2_O_2_ (Brander et al., 2021). This led to initial assay conditions of: 100 nM Cu(II)-LPMO, 6.35 μM rFl, 100 μM DHA, and 100 μM H_2_O_2_ in a citrate–phosphate buffer at pH 7.24. This resulted in a clear time-and enzyme-concentration-dependent increase in Fl fluorescence (Figure 2A). An important control in which CuCl_2_ was used instead of the enzyme showed that “free” Cu(II) did not cause any increase in fluorescence, and that Cu(II) without DHA or H_2_O_2_ did not lead to the development of fluorescence (Supplementary Figure S1B). The background signal from water blanks was negligible. The rFl oxidation rate was faster with *Ta*AA9A than with *Ls*AA9A, and continued optimization of the assay was therefore performed with *Ta*AA9A. The sensitivity of the assay was tested using a 0–1 μM gradient of *Ta*AA9A and the progress curves are shown in Figure 2B. The initial rates of the reaction as a function of the concentration of *Ta*AA9A showed a linear dependency throughout the concentration range (Figure 2C). Notably, small standard errors of the progress curves and conversely small standard errors in the calculated apparent rates are observed. As expected, the assay is dependent on the holo-state of the enzyme; the activity increasing with concentration and leveling off after 1:1 stoichiometry (data not shown). The assay showed linearity with regard to the rFl substrate in the range 6.35–19 μM, as demonstrated by the total oxidation of rFl with HRP and comparison with a standard curve of Fl (Supplementary Figure S1A).

### 3.1. Cu(II)-LPMOs and HRP showed activity in the assay

To investigate the specificity of the rFl-based enzyme activity assay, a selection of different LPMOs was tested in parallel with a small series of different oxidoreductases. Three different LPMO families, AA9, AA10, and AA13 were included together with CopC, a redox inert copper chaperone, that function as a negative control and horseradish peroxidase (HRP) that was expected to be a positive control (Figure 2D). Clearly, Cu(II)-LPMOs are able to oxidize rFl in the presence of DHA and H_2_O_2_. The other oxidoreductases were tested at the relatively high concentration of 1 μM to ensure that even low levels of rFl oxidizing activity would be detected. Flavin oxidase, superoxide dismutase, cytochrome P450 oxidase, galactose oxidase and three laccases were tested and all activities were less than that of 30 nM *Nc*AA9E, the worst performing LPMO (Supplementary Figure S1C). Laccases can oxidize a wide range of phenolic compounds, but their reaction in the rFl assay was modest. The low activity can be caused by the neutral pH that is known to inhibit the enzyme class (Xu et al., 1996). HRP exhibits a faster oxidation rate than the Cu(II)-LPMOs, but to our surprise, HRP does not strictly require H_2_O_2_ as a co-substrate to oxidize rFl to Fl. The HRP activity is only slightly enhanced by the addition of DHA (Supplementary Figure S1D).

This level of enzyme specificity could potentially be of advantage when LPMOs are to be purified from fermentation broths. For an initial test of such an application of the assay, 0–3 μM Cu(II)-*Ta*AA9A was diluted in LB media or milli Q water. LB media decreased the rFl oxidation rate but the activity was clearly above the assay background with enzyme concentrations as low as 75 nM Cu(II)-*Ta*AA9A (Supplementary Figure S1E).

### 3.2. Dose–response of DHA, H_2_O_2_ and ascorbate

**Figure 3:**
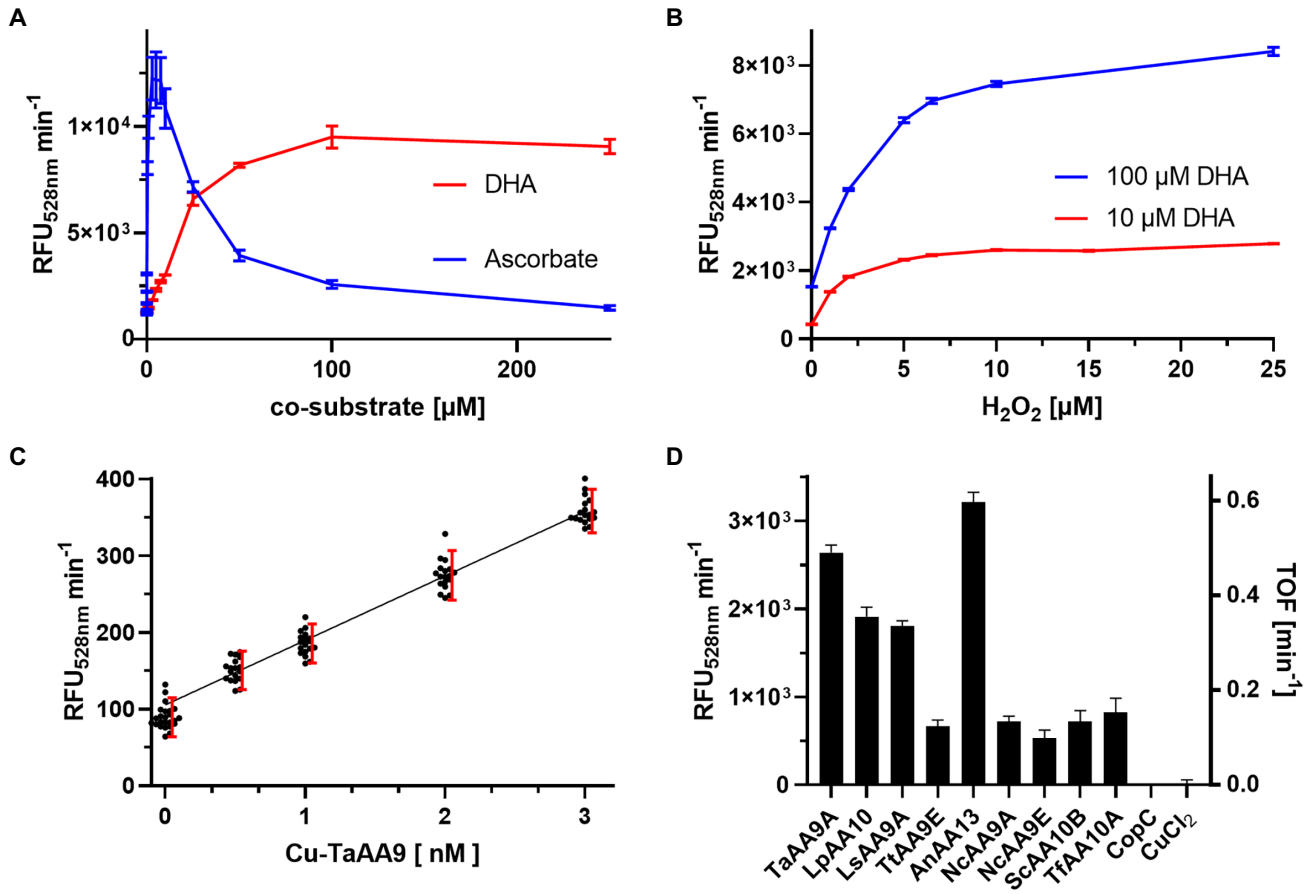
Dose response of the rFl assay **(A)** Initial rates of rFl oxidation by 30 nM Cu(II)-*Ta*AA9A with 6.35 μM rFl, 100 μM H_2_O_2_ and 0–250 μM DHA (red) or ascorbate (blue). **(B)** Initial rates of rFl oxidation by 30 nM Cu(II)-*Ta*AA9A with 6.35 μM rFl, 0–25 μM H_2_O_2_ and 10 (red) or 100 (blue) μM DHA (See also Supplementary Figure S2). **(C)** Initial rates of rFl oxidation by 0–3 nM Cu-Ta with 10 μM DHA, 10 μM H_2_O_2_ and 6.35 μM rFl. Each enzyme concentration was prepared as 18-fold redundant samples. Red bars demarks the 90% normal distribution range and the black line is a linear fit of data from the range of 0.5–3 nM Cu-Ta. **(D)** The initial rates of a series of 30 nM Cu(II)-LPMOs with 10 μM DHA and 10 μM H_2_O_2_.

DHA and ascorbate were tested individually in a concentration-dependent manner to determine their effect on enzyme activity. Figure 3A shows the enzymatic rates of Fl production by 30 nM Cu(II)-*Ta*AA9A in presence of 0–250 μM DHA or 0–250 μM ascorbate and 100 μM H_2_O_2_. Addition of DHA enhances the enzyme activity by a factor of 100, and the full effect is realized at 100 μM DHA with only a small diminishing effect of adding more. Addition of ascorbate also enhances the enzyme activity to a similar level, but the full effect is reached at 3–7.5 μM and adding more gives a diminishing effect (Supplementary Figure S2). Ascorbate is a known co-substrate for LPMO activity, but it can interfere with the LPMO assays through radical scavenging, H_2_O_2_ formation and even enzyme autooxidation (Schwaiger et al., 2023). DHA has not been shown to have similar interferences and was chosen as the preferred co-susbstrate. The dose response of DHA is linear in the range of 0–25 μM and to investigate the H_2_O_2_ dependency of rFl production, reactions with either 10 or 100 μM DHA were tested (Figure 3B). A rapid increase in reaction rates were observed with increasing concentrations of H_2_O_2_, leading to a wide plateau between 6.5 and 250 μM H_2_O_2_ (Supplementary Figure S2B).

rFl can be be bought from commercial vendors under the trivial name or CAS number 518–44-5 (See Supplementary Figure S3).

### 3.3. The rFl assay works at surprisingly low concentrations of H_2_O_2_

The low concentration optima for both ascorbate and H_2_O_2_, supports an activity where the concentration of H_2_O_2_ must be kept low to be advantageous and not detrimental to enzyme activity (Brander et al., 2021; Stepnov et al., 2021). We chose to explore the assay performance in this low concentration regime. The sensitivity was tested in a series of reactions with 0–10 nM Cu(II)-*Ta*AA9A, 10 μM DHA and 10 μM H_2_O_2_. Each enzyme concentration was tested in 18 individual experiments, and the rate of fluorescence increase shows a linear correlation (Figure 3C). Breslmayr et al. (2018) and Brander et al. (2021) have used the lack of overlap between 90% of the normal distributed data sets (*σ* = 1.645) from samples with or without enzyme as the threshold for assay sensitivity. These limits are visualized in the dose response plot and show that the limit of detection (LOD) in the rFl assay is 0.5 nM Cu-*Ta*AA9A. However, the blank is slightly below the linear dose response curve and will overestimate the quantification limit. The assay cannot distinguish between 0.5 nM and 1.0 nM Cu-*Ta*AA9A and more reasonable assessment of sensitivity is that limit of quantification (LOQ) of the rFl assay is 1 nM Cu-*Ta*AA9A. We repeated the screening of LPMOs in the lower concentration range, and found a relatively similar assay response as in the concentrated samples (Figure 3D).

## 4. Discussion

### 4.1. The assay is sensitive, reproducible, and easy to adapt

An LPMO catalyzed reaction between rFl and H_2_O_2_ has been demonstrated and used to develop an enzyme activity assay. The product is the stable and fluorescent Fl compound and the reaction can be monitored continuously by fluorescence spectrophotometry. The rFl compound was produced in the laboratory by boiling Fl with solid zinc followed by neutralization and filtration. A commercial preparation of rFl was tested and gave similar reaction rates, but the larger background level complicated analysis.

The rFl assay was found to be a sensitive probe for LPMO activity with a sensitivity of 1 nM of *Ta*AA9A. The assay has a linear dose response in the concentration range of 1–1,000 nM. Breslmayr et al. has reported an LPMO sensing DMP peroxidase assay that can be measured continuously by absorption spectrophotometry and is linear in the concentration range of 12.5–1,000 nM *Nc*AA9C (Breslmayr et al., 2018). The low detection limit of the rFl assay can probably be attributed to the very low background signal of the fluorescent system. Another advantage of the rFl assay is that the product is stable and the reaction time can be extended if necessary. This is in contrast to the DMP system that is prone to over oxidization and formation of insoluble polymers (Breslmayr et al., 2019). A third advantage of the rFl system is that the fluorogenic nature of the product makes the assay useful in opaque solution as demonstrated by verification of 75 nM *Ta*AA9A in LB medium.

### 4.2. RFl is also a peroxidase substrate

HRP showed assay activity in the same range as LPMOs and it is possible that the rFl assay can also be used to determine peroxidase activity. Almost by definition, HRP uses H_2_O_2_ to oxidize its substrates, but surprisingly, H_2_O_2_ was not required for the oxidation of rFl. This suggests that the rFl assay can differentiate between the enzyme classes. However, for the most accurate results, it is recommended to use the assay when LPMOs are the primary contributors to oxidase activity. The mechanism by which the two enzyme classes differ is beyond the scope of this study and will require further research.

### 4.3. The effect of DHA and ascorbate on the assay

The LPMO dependent oxidation of rFL was enhanced by 100 μM DHA which is similar to the effect reported on rPh oxidation (Brander et al., 2021). The rPh assay was formulated so that the DHA enhancing effect was specific to LPMO activity. A similar specificity was not seen for the rFl assay. The difference might be attributed to the nanomolar sensitivity of the assay where small impurities of the enzymes preparation can carry over in the assay samples. The rFl assay response was also greatly enhanced by addition of ascorbate with an optimal concentration around 5 μM. This is another difference to the rPh assay where ascorbate was strictly inhibiting. It has previously been shown that LPMOs can rapidly oxidize ascorbate in a reaction with H_2_O_2_ (Brander et al., 2021) and to avoid interference by this activity, the rFl-based LPMO activity assay was formulated with DHA. Due to the low level of ascorbate needed to enhance the reaction, it is likely to react with the enzyme rather than the other substrates. This suggest that the rFl oxidation is catalyzed by the reduced state, Cu(I)-LPMO, similar to other suggested mechanisms (Bissaro et al., 2017; Stepnov et al., 2022). It is possible that the effect of DHA addition also is to reduce the active site copper (Jung and Wells, 1998).

### 4.4. Hydrogen peroxide

The rFl oxidation was found to increase with H_2_O_2_ until a plateau was observed at 6–250 μM. The onset of the plateau is at a concentration similar to the 6.35 μM rFl used in the assay, and this indicates that the oxidation of rFl can be coupled to consumption of H_2_O_2_. The stoichiometry between rFl and H_2_O_2_ together with the enabling role of reductants suggests a reaction mechanism similar to the peroxidase driven mechanism first suggested by Bissaro et al. (2017) (See Supplementary Figure S4). The width of the plateau shows resilience against autoxidation with H_2_O_2_ and can probably be attributed to the stability of the used model LPMO, *Ta*AA9A. This enzyme has previously been shown to tolerate 200 μM H_2_O_2_ (Petrović et al., 2018) when in reaction with cellulose substrate. *Ls*AA9A has been shown to tolerate 80 μM H_2_O_2_ when in reaction with soluble cellulose-oligosaccharides (Brander et al., 2021) and CBP21 has been reported to tolerate 20 μM H_2_O_2_ in reaction with chitin (Kuusk et al., 2018). These tolerance levels are likely to decrease in an assay mixture that does not include the natural LPMO substrate, and thus the rFl assay was optimized at the low concentration of 10 μM H_2_O_2_. The DMP assay use 100 μM H_2_O_2_ and we suggest that the lower level of H_2_O_2_ in the rFl assay makes it preferable for assaying LPMOs.

## 5. Conclusion

A protocol has been developed for a fast and sensitive LPMO activity assay based on enzymatic oxidation of rFl into Fl. The reactivity of different LPMOs toward rFl varies. The assay has a sensitivity range of 1 nM –1,000 nM LPMO and uses a relatively low concentration of just 10 μM H_2_O_2_. This LPMO activity assay may be useful for screening purposes when the H_2_O_2_ sensitivity of an LPMO is not yet established, and for detailed characterization of individual LPMOs.

## Data availability statement

The original contributions presented in the study are included in the article/Supplementary material, further inquiries can be directed to the corresponding author.

## Author contributions

SB conceptualized the rFl to Fl oxidation and its use as a LPMO-specific substrate. JI performed the experiments. KJ oversaw the study. All authors contributed to the article and approved the submitted version.

## Funding

This study was supported by the Novo Nordisk Foundation, grant nos. NNF17SA0027704 and NNF20OC0059697 to KJ.

## Conflict of interest

The authors declare that the research was conducted in the absence of any commercial or financial relationships that could be construed as a potential conflict of interest.

## Publisher’s note

All claims expressed in this article are solely those of the authors and do not necessarily represent those of their affiliated organizations, or those of the publisher, the editors and the reviewers. Any product that may be evaluated in this article, or claim that may be made by its manufacturer, is not guaranteed or endorsed by the publisher.
