# Supplementary material for: A fast, sensitive and fluorescent LPMO activity assay
Source: Front Microbiol. 2023 Mar 14;14:1128470. doi: 10.3389/fmicb.2023.1128470 (PMC10043361; doi:10.3389/fmicb.2023.1128470)
Supplement: Supplementary file 1 [file Data_Sheet_1.docx]

**A fast, sensitive and fluorescent LPMO activity assay**

**Johan Ø. Ipsen, Katja S. Johansen, Søren Brander*^1*^***

^1^Department of Geosciences and Natural Resource Management, University of Copenhagen, DK-1958 Copenhagen, Denmark

**Correspondence:**^*^Corresponding Author
sbd@ign.ku.dk

Supplementary Information

**Supplementary Figure S1**. **A)** Standard curve of Fl with a linear regression (dotted line) of r^2^ = 0.977. Highlighted data points are 6.35 µM (green), 12.7 µM (blue) and 19.05 µM (red) of rFl oxidized by HRP. **B)** Progress curves showing lack of rFl oxidation by 1 µM CuCl_2_ with H_2_O_2_ (cyan), DHA (magenta) or both (orange). **C)** Initial rates of rFl oxidation by several oxidoreductases at 1 µM with 6.35 µM rFl, 100 µM DHA, and 100 µM H_2_O_2_ in citrate-phosphate buffer at pH 7.25. The rates are at least two orders of magnitude lower than for *Ta*AA9A **D)** Initial rates of rFl oxidation by HRP with 6.35 µM rFl, 0-100 µM H_2_O_2_ with (blue) or without (red) 100 µM DHA. **E)** Dose response of Cu-*Ta*AA9A prepared in milli Q water (red) or LB medium (blue). After dilutions, the assay mixture consist of 0-0.75 µM Cu-*Ta*AA9A, 6.35 µM rFl, 100 µM DHA, 100 µM H_2_O_2_, 50 mM citrate-phosphate buffer at pH 7.25 and 0 or 25% LB medium. Fluorescence is generated above the background level at 75 nM Cu(II)-*Ta*AA9A.

**Supplementary Figure 2.** Apparent rates of rFl oxidation **A)** Zoom in on the data presented in the main text Figure 3A. Initial rates of rFl oxidation by 30 nM Cu(II)-*Ta*AA9A with 6.35 µM rFl, 100 µM H_2_O_2_ and 0 –10 µM DHA (red) or ascorbate (blue). A plateau is observed in the range 3-7.5 µM H_2_O_2_. **B)** Zoom out on the data presented in the main text Figure 3B. Initial rates of rFl oxidation by 30 nM Cu(II)-*Ta*AA9A with 6.35 µM rFl, 0 –1 mM H_2_O_2_ and 10 (red) or 100 (blue) µM DHA.

**Supplementary Figure 3.** Comparison of commercially available and in-house prepared rFl. Commercial rFl was acquired from Merck, CAS 518-44-5. The powder was wetted in a few drops of ethanol and diluted to 5 mM in milli Q water. The in-house rFl was prepared fresh, according to protocol in the main text. **A)** Reactions of 100 nM Cu-*Ta*AA9A, 100 µM DHA, 100 µM H_2_O_2_, 25 mM citrate-phosphate buffer at pH 7.25 and 0.625, 1.25, 6.25, 12.5 µM of in-house (red) and commercial (blue) rFl. **B)** Data from A) plotted as the reaction rates against starting concentration of rFl. The two sources of rFl behaves similarly, but the preparation of commercial rFl shows a larger background signal that complicates estimation of reaction rates from initial slopes of kinetic traces.


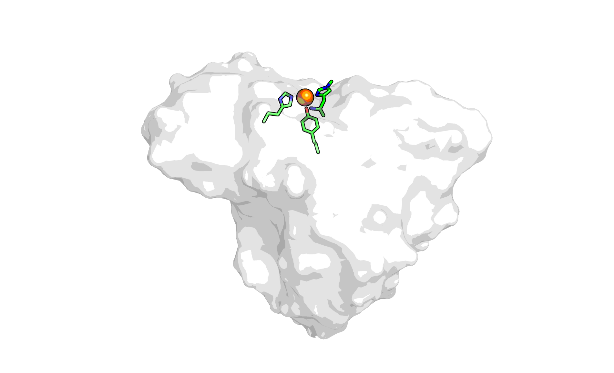

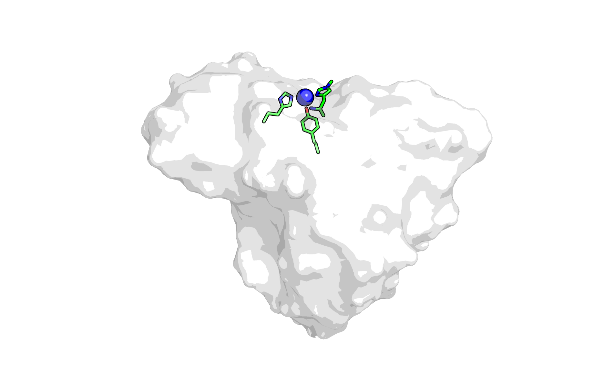


**rFl**

**Fl**

**Supplementary Figure 4.** Diagram of an LPMO catalyzed oxidation of rF. Data reported in the main supports a reaction mechanism where the LPMO is first reduced to Cu(I)-LPMO and it this form that catalyze oxidation of rFl and stoichiometrically consumption of H_2_O_2_.
